# Two-Step Calibration Method for Inverse Finite Element with Small Sample Features

**DOI:** 10.3390/s20164602

**Published:** 2020-08-16

**Authors:** Libo Xu, Feifei Zhao, Jingli Du, Hong Bao

**Affiliations:** Key Laboratory of Electronic Equipment Structure Design of Ministry of Education, Xidian University, Xi’an 710071, China; xulibo@stu.xidian.edu.cn (L.X.); ffzhao@stu.xidian.edu.cn (F.Z.); jldu@mail.xidian.edu.cn (J.D.)

**Keywords:** non-uniform rational B-spline, inverse finite element, deformation reconstruction, fuzzy network

## Abstract

When the inverse finite element method (inverse FEM) is used to reconstruct the deformation field of a multi-element structure with strain measurements, strain measurement errors can lower the reconstruction accuracy of the deformation field. Furthermore, the calibration ability of a self-structuring fuzzy network (SSFN) is weak when few strain samples are used to train the SSFN. To solve this problem, a novel two-step calibration method for improving the reconstruction accuracy of the inverse FEM method is proposed in this paper. Initially, the errors derived from measured displacements and reconstructed displacements are distributed to the degrees of freedom (DOFs) of nodes. Then, the DOFs of nodes are used as knots, in order to produce non-uniform rational B-spline (NURBS) curves, such that the sample size employed to train the SSFN can be enriched. Next, the SSFN model is used to determine the relationship between the measured strain and the DOFs of the end nodes. A loading deformation experiment using a three-element structure demonstrates that the proposed algorithm can significantly improve the accuracy of reconstruction displacement.

## 1. Introduction

With the development of health monitoring and intelligent structures, structural deformation sensing technology based on strain measurement data has become increasingly important [1]. The accurate deformation reconstruction of plates, beams, and other structures provides a basis for ensuring the safe operations of aircraft. As a result, it is of great significance to achieve high-precision structural deformation reconstruction.

The key to deformation reconstruction is constructing a relationship between the structural deformation and strain measurements. Many research methods have been proposed by domestic and foreign scholars for this purpose, such as the modal transformation method, the Ko method, and inverse FEM. Among them, the inverse FEM is an accurate and effective method for deformation reconstruction. The inverse FEM was first proposed by Tessler et al., who employed a variational principle based on a least-squares functional [2,3]. The modal transformation method can accurately reconstruct the deformation of a plate and beam structure [4], but it needs an accurate finite element model. The Ko method is based on the classical Euler–Bernoulli beam theory [5]. It integrates discrete surface strain measurements with piecewise-continuous polynomials to achieve high-precision reconstruction of a beam element in a one-dimensional direction. The triangle element inverse FEM method was proposed to reconstruct the structural deformation in real-time by measuring the strain, based on the first-order shear deformation theory [6,7], and it solved the problem of the Ko and modal methods, in that they cannot adapt to complex topological structures and boundary conditions. Compared with the Ko method and the modal transformation method, the triangular element inverse FEM method has high reconstruction accuracy without considering the material properties and load information of the structure, and it can quickly reconstruct the structure deformation. However, this method presents pathological behavior, which has been labeled as shear-locking, in membrane reconstruction. Subsequently, the inverse FEM of the quadrilateral elements method was proposed by Kefal and Oterkus et al. [8,9]. The proposed method considers the degree of freedom of rotation in the Z direction, thus avoiding singular matrices during the inversion process and overcoming the shear-locking phenomenon in the deformation reconstruction of membranes. However, in engineering, with the application of composite structures, the inverse finite element method based on shear deformation theory was not suitable for the deformation reconstruction of composite structures. Cerracchio et al. improved the original inverse FEM formulation by adding the kinematic assumptions of the Refined Zigzag Theory [10]. This method is suitable for the displacement monitoring of multi-layer composite materials and sandwich structures with high anisotropy and heterogeneity. Meanwhile, Gherlone et al. proposed the inverse FEM of beams, considering the problems of beam stretching, bending, and torsional deformation [11,12].

In practical engineering, the installation position of the sensor greatly affects the reconstruction accuracy. In [13], a model update method was proposed based on the measured values of sensors installed at arbitrary positions and orientations. This method can establish the model independently, regardless of the position of the sensor, but it requires more model information. In [14,15], an optimized sensor layout model based on eigenvalue analysis was proposed, in order to improve the robustness of the reconstruction model. In [16], Islam et al. proposed a robust object tracking method based on a dynamic learning rate. This method uses a transformation filter based on the kernel correlation function filter, which improves the robustness of the model. In [17], a double objective optimization model was established, considering both accuracy and robustness in the reconstruction of beams by using the inverse finite element method. It improved the reconstruction accuracy and robustness. In [18], Kim et al. proposed a sequential framework for improving the recognition ability of FEM models. The proposed method could update the initial FE model accurately and improve the reliability of prediction performance. In [19], Khatir et al. proposed an effective target recognition method that used the Jaya algorithm to improve the training of artificial neural network parameters and improved the accuracy of target data prediction. However, this method has the risk of losing information. In [20], Pan approximated the relationship between the measured strain and the inverse solution strain by using the SSFN. Using 13 sets of working condition data, the fuzzy calibration network was established. Then, the calibration strain was used to solve for the deformation displacement of the frame. In [21], a fuzzy calibration network with support vectors was proposed, training 10 sets of working condition data. However, in the above literature, only a small amount of data was used to train the correction network, which directly affects the accuracy of the network for insufficiently covered information in the established network.

In this paper, a two-step calibration method is proposed for shape sensing with small sample features. The proposed method is used to calibrate the reconstruction displacement and improve the accuracy of deformation reconstruction.

Briefly, this paper is organized as follows: In Section 2, two problems in deformation reconstruction are explained. One is that the displacement of the end element of a multi-element structure is affected by the angle deflection of the front element, which can lead to a large reconstruction error. Second, if the different working condition data that can be collected has a small sample size, the reconstruction accuracy can be seriously affected. In Section 3, the reconstruction displacement is separated, and the error distribution is processed. Then, the sample data are expanded by the NURBS method and used to construct SSFN. Finally, in Section 4, experiments are performed on a three-element frame, in order to verify the effect of the proposed calibration algorithm on improving the accuracy of deformation reconstruction.

## 2. Multi-Element Displacement Analysis

**Figure 1:**
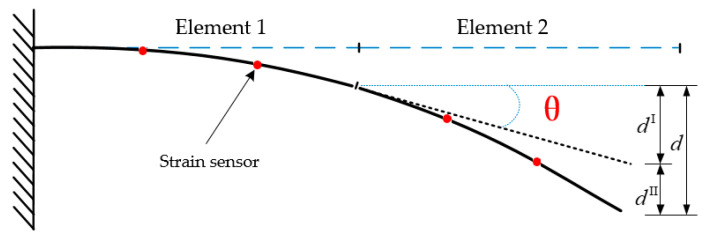
Structural deformation and displacement.

Strain sensors are placed on the surface of a multi-element frame to measure its surface strain, as shown in Figure 1. Then, inverse FEM can be used to reconstruct the structural deformation displacement of the multi-element frame, using the collected strain. The displacements are composed of two parts: one is elastic displacement, and the other is the projection displacement, which is caused by rotation of the first element.

The structural deformation can be expressed as

(1) $$d=d^{I}+d^{II},$$

where d represents the structural deformation displacement; $d^{I}$ denotes the projected displacement, which is independent of strain measurement and related to rotation; and $d^{II}$ is the elastic displacement of the structure, which is determined only by the strain. Closer to the end of the frame, the proportion of elastic displacement in the frame displacement decreases rapidly, such that the final element’s displacement is almost composed solely of the projected displacement (as shown in Figure 1). This makes direct calibration poor. In addition, in some special experimental environments, less experimental data can be collected, such that calibration capability of the SSFN algorithm is decreased.

The source of the reconstruction error can be analyzed. On the one hand, inaccurately placed strain sensor positions can cause installation errors; on the other hand, the strain measurement system can induce measurement errors. Together, these factors cause strain errors which, in turn, affect the reconstruction accuracy. Therefore, calibration plays an important role in high-precision deformation reconstruction.

## 3. Establishing a Small Sample Fuzzy Calibration Model

The calibration process is divided into three parts: initially, the elastic displacement is separated, according to inverse FEM knowledge, and the elastic displacement errors are assigned to the kinematics variables $u^{e}={\left[u,v,w,\theta_{x},\theta_{y},\theta_{z}\right]}^{T}$.

Then, the kinematics variables are used to solve the NURBS curve, such that the sample size of strain errors is expanded. Finally, the SSFN model is established using the extended data, which approximates the relationship between the measured strain and the corrected displacement, in order to improve the accuracy of deformation reconstruction.

### 3.1. The First Step of Calibration: Deformation Separation and Error Distribution

Consider the phenomenon that the closer the evaluated position is to the fixed end, the smaller the elastic displacement is; the elastic displacement is separated for correction in each element in this paper. According to the inverse FEM theory, the strain–displacement relationship can be presented as follows:

(2) KU=F,

where the matrix K is determined by the measured position of the strain and is independent of the strain data, F resembles the load vector, and U comprises the nodal degrees of freedom [22]. Equation (2) can be partitioned as:

(3) $$\left[\begin{array}{ccc} K_{11} & K_{12} & K_{13}\\ K_{21} & K_{22} & K_{23}\\ K_{31} & K_{32} & K_{33} \end{array}\right]\left\{\begin{array}{c} U_{1}\\ \begin{array}{c} U_{2}\\ U_{O} \end{array} \end{array}\right\}=\left\{\begin{array}{c} F_{1}\\ \begin{array}{c} F_{2}\\ F_{3} \end{array} \end{array}\right\},$$

where $U_{1}$ is the degree of freedom of the starting point, $U_{2}$ is the degree of freedom of the end node, and $U_{O}$ is the vector of internal degrees of freedom. For a cantilever beam, according to Equation (3), the reconstructed elastic displacement ($d_{iFEM}^{II}$) of the end point is

(4) $$d_{iFEM}^{II}=U_{2}={\left(K_{22}-K_{23}K_{13}^{-1}K_{12}\right)}^{-1}\left[F_{2}+\left(K_{23}K_{13}^{-1}K_{11}-K_{21}\right)U_{1}-K_{23}K_{13}^{-1}F_{1}\right].$$

When the starting point degree of freedom is $U_{1}=0$, Equation (4) is suitable for solving the elastic displacement of the first element; when $U_{1}$ is not equal to **0**, it is suitable for other elements.

In addition, when $F_{1}=F_{2}=F_{3}=0$, the resulting projection displacement $d^{I}$ can be calculated by Equation (3):

(5) $$d^{I}=U_{2}^{\prime }={\left(K_{22}-K_{23}K_{13}^{-1}K_{12}\right)}^{-1}\left(K_{23}K_{13}^{-1}K_{11}-K_{21}\right)U_{1}.$$

When the starting point degree of freedom is $U_{1}=0$, Equation (5) is suitable for solving the projection displacement of the first element; when $U_{1}$ is not equal to 0, it is suitable for other elements.

Then, in the actual experimental measurements, the measured displacement can be denoted by d. So, we can use Equation (5) to calculate the actual elastic displacement ($d_{act}^{II}$) as

(6) $$d_{act}^{II}=d-d^{I}.$$

Therefore, the elastic displacement reconstruction errors can be obtained as

(7) $$\{\begin{array}{c} \Delta u_{x}\left(x_{i},y_{i},z_{i}\right)=d_{{act}_{x}}^{II}\left(x_{i},y_{i},z_{i}\right)-d_{{iFEM}_{x}}^{II}\left(x_{i},y_{i},z_{i}\right)\\ \Delta u_{y}\left(x_{i},y_{i},z_{i}\right)=d_{{act}_{y}}^{II}\left(x_{i},y_{i},z_{i}\right)-d_{{iFEM}_{y}}^{II}\left(x_{i},y_{i},z_{i}\right)\\ \Delta u_{z}\left(x_{i},y_{i},z_{i}\right)=d_{{act}_{z}}^{II}\left(x_{i},y_{i},z_{i}\right)-d_{{iFEM}_{z}}^{II}\left(x_{i},y_{i},z_{i}\right), \end{array}$$

where $\Delta u_{x}\left(x_{i},y_{i},z_{i}\right)$, $\Delta u_{y}\left(x_{i},y_{i},z_{i}\right)$, and $\Delta u_{z}\left(x_{i},y_{i},z_{i}\right)$ represent the elastic displacement errors in the X, Y, and Z directions at the end point $\left(x_{i},y_{i},z_{i}\right)$, respectively. In the same way, the elastic displacement errors $\Delta u_{ox}\left(x_{o},y_{o},z_{o}\right)$, $\Delta u_{oy}\left(x_{o},y_{o},z_{o}\right)$, and $\Delta u_{oz}\left(x_{o},y_{o},z_{o}\right)$ of an internal node can be obtained.

Next, the error distribution can be derived. During the reconstruction process, the displacement of any point in an element can be defined by the displacement of the nodes (the end node and an internal node) and the shape function (see Appendix A). Therefore, when the elastic displacement is calibrated, the displacement error needs to be distributed to two nodes. According to the inverse finite element deformation field theory [23,24], the node displacement errors can be distributed as follows:

(8) $$\{\begin{array}{l} \Delta u_{x}\left(x_{i},y_{i},z_{i}\right)=\Delta u\left(x_{i}\right)+z_{i}\Delta \theta_{y}\left(x_{i}\right)-y_{i}\Delta \theta_{z}\left(x_{i}\right)\\ \Delta u_{y}\left(x_{i},y_{i},z_{i}\right)=\Delta v\left(x_{i}\right)-z_{i}\Delta \theta_{x}\left(x_{i}\right)\\ \Delta u_{z}\left(x_{i},y_{i},z_{i}\right)=\Delta w\left(x_{i}\right)+y_{i}\Delta \theta_{x}\left(x_{i}\right), \end{array}$$

where, $\Delta u\left(x_{i}\right),\;\Delta v\left(x_{i}\right),\;\Delta w\left(x_{i}\right),\;\Delta \theta_{x}\left(x_{i}\right),\Delta \theta_{y}\left(x_{i}\right),$ and $\Delta \theta_{z}\left(x_{i}\right)$ represent the error distribution values of the six kinematic variables of the end node (i.e., the solution values of the distribution algorithm). In the same way, the error distribution results of internal nodes $\Delta u_{o}\left(x_{o}\right)$, $\Delta v_{o}\left(x_{o}\right)$, $\Delta w_{o}\left(x_{o}\right)$, $\Delta \theta_{ox}\left(x_{o}\right)$, $\Delta \theta_{oy}\left(x_{o}\right),$ and $\Delta \theta_{oz}\left(x_{o}\right)$ can be obtained.

The principle of error distribution is defined as follows:

(9) $$\{\begin{array}{l} \Delta v\left(x_{i}\right)=\;\frac{j}{100}\Delta u_{y}\left(x_{i},y_{i},z_{i}\right)\\ \Delta u\left(x_{i}\right)=\frac{m}{100}\Delta u_{x}\left(x_{i},y_{i},z_{i}\right)\\ \Delta \theta_{y}\left(x_{i}\right)=\;\frac{n}{100z_{i}}\Delta u_{x}\left(x_{i},y_{i},z_{i}\right), \end{array}$$

where j,m,n∈[0,100]. Then, according to Equation (8), $\Delta \theta_{x}\left(x_{i}\right)$, $\Delta w\left(x_{i}\right)$, and $\Delta \theta_{z}\left(x_{i}\right)$ can be obtained:

(10) $$\{\begin{array}{l} \Delta \theta_{x}\left(x_{i}\right)=\;\frac{j-100}{100z_{i}}\Delta u_{y}\left(x_{i},y_{i},z_{i}\right)\\ \Delta w\left(x_{i}\right)=\Delta u_{z}\left(x_{i},y_{i},z_{i}\right)+\frac{100-j}{100z_{i}}\Delta u_{y}\left(x_{i},y_{i},z_{i}\right)\\ \Delta \theta_{z}\left(x_{i}\right)=\;\left(m+n-1000\right)\frac{\Delta u_{x}\left(x_{i},y_{i},z_{i}\right)}{100y_{i}}. \end{array}$$

When a set of values (j,m,n) is arbitrarily combined into Equations (9) and (10), a set of corrected node displacements can be obtained, and the displacement can be further obtained using the displacement shape function. In order to further evaluate the accuracy of the calibrated displacement, the root mean square error (RMSE) is used as the evaluation index:

(11) $$RMSE_{1}=\sqrt[{2}]{\frac{\overset{n}{\underset{i=1}{\sum }}{\left(d_{act}^{II}\left(x_{i},y_{i},z_{i}\right)-d_{mod}^{II}\left(x_{i},y_{i},z_{i}\right)\right)}^{2}}{n},}$$

where $d_{mod}^{II}\left(x_{i},y_{i},z_{i}\right)$ is the corrected value of the elastic displacement.

In order to better describe the error distribution process, the form of pseudo code is adopted, as shown in Algorithm 1:
**Algorithm 1.** Error distribution algorithm.**Input: (1)** Elastic displacement error $\Delta u\left(x_{i},y_{i},z_{i}\right),\;\Delta u_{y}\left(x_{i},y_{i},z_{i}\right),\;\Delta u_{z}\left(x_{i},y_{i},z_{i}\right)$,$\;\Delta u_{0x}\left(x_{o},y_{o},z_{o}\right)$    **(2)** Actual elastic displacement $d_{act}^{II}\left(x_{i},y_{i},z_{i}\right)$**Output:** Error distribution result $\Delta u\left(x_{i}\right)$,$\Delta v\left(x_{i}\right)$, $\Delta w\left(x_{i}\right)$,$\;\Delta \theta_{x}\left(x_{i}\right)$,$\;\Delta \theta_{y}\left(x_{i}\right)$,$\;\Delta \theta_{z}\left(x_{i}\right)$,$\;\theta_{0y}\left(x_{o}\right)$,$\;\theta_{0z}\left(x_{o}\right)$01:  Begin02:  Input  $\;\Delta u_{x}\left(x_{i},y_{i},z_{i}\right)$, $\Delta u_{y}\left(x_{i},y_{i},z_{i}\right)$, $\Delta u_{z}\left(x_{i},y_{i},z_{i}\right)$, $d_{act}^{II}\left(x_{i},y_{i},z_{i}\right)$03:  Initial  $\;j=0,m=0,n=0,\;\;RMSE_{1}\_min$ = 100,00004:  for j = 0 to 100 step 105:   $\;\Delta v\left(x_{i}\right)=\left(j/100\right)\Delta u_{y}\left(x_{i},y_{i},z_{i}\right)$06:   $\;\Delta \theta_{x}\left(x_{i}\right)$ = $\left(\Delta u_{y}\left(x_{i},y_{i},z_{i}\right)\;-\;\Delta v\left(x_{i}\right)\right)/\left(-z_{i}\right)$07:   $\;\Delta w\left(x_{i}\right)$=$\Delta u_{z}\left(x_{i},y_{i},z_{i}\right)\;-y_{i}\cdot \Delta \theta_{x}\left(x_{i}\right)$08:    for m = 0 to 100 step 109:    $\Delta u\left(x_{i}\right)$=$\left(m/100\right)\cdot \;\Delta u_{x}\left(x_{i},y_{i},z_{i}\right)$10:     for n = 0 to 100 step 111:     $\Delta \theta_{y}\left(x_{i}\right)$=$\left(\left(n/100\right)\cdot \;\Delta u_{x}\left(x_{i},y_{i},z_{i}\right)\right)/z_{i}$12:     $\Delta \theta_{y}\left(x_{i}\right)$=$\left(\left(n/100\right)\cdot \;\Delta u_{x}\left(x_{i},y_{i},z_{i}\right)\right)/z_{i}$13:     $\Delta \theta_{z}\left(x_{i}\right)$=$\left(\left(n+m-100\right)\cdot \;\Delta u_{x}\left(x_{i},y_{i},z_{i}\right)\right)/\left(100\cdot y_{i}\right)$14:     $\Delta \theta_{oy}\left(x_{i}\right)$=$\left(\left(n/100\right)\cdot \;\Delta u_{ox}\left(x_{i},y_{i},z_{i}\right)\right)/z_{i}$15:     $\Delta \theta_{oz}\left(x_{i}\right)$=$\left(\left(n+m-100\right)\cdot \;\Delta u_{ox}\left(x_{i},y_{i},z_{i}\right)\right)/\left(100\cdot y_{i}\right)$16:     $d_{mod}^{II}\left(x_{i},y_{i},z_{i}\right)$
$\overset{\;\mathrm{Shape}\;\mathrm{function}\;}{\leftarrow }$ [$\Delta u\left(x_{i}\right),\Delta v\left(x_{i}\right),\;\Delta w\left(x_{i}\right),\;\Delta \theta_{x}\left(x_{i}\right),\;\Delta \theta_{y}\left(x_{i}\right),\Delta \theta_{z}\left(x_{i}\right)$]17:     $RMSE_{1}=\sqrt[{2}]{\frac{\overset{n}{\underset{i=1}{\sum }}{\left(d_{act}^{II}\left(x_{i},y_{i},z_{i}\right)-d_{mod}^{II}\left(x_{i},y_{i},z_{i}\right)\right)}^{2}}{n}}$18:     if $RMSE_{1}$ < $RMSE_{1}\_min$19:      $RMSE_{1}\_min\overset{}{\leftarrow }RMSE_{1}$20:       $result\overset{}{\leftarrow }$ [$\Delta u\left(x_{i}\right),\Delta v\left(x_{i}\right),\;\Delta w\left(x_{i}\right),\;\Delta \theta_{x}\left(x_{i}\right),\;\Delta \theta_{y}\left(x_{i}\right),\Delta \theta_{z}\left(x_{i}\right),\;\theta_{0y}\left(x_{o}\right),\;\theta_{0z}\left(x_{o}\right)$]21:      end22:    Next *n*23:   Next *m*24:  Next j25:  Return result26:  End


### 3.2. Sample Extension

In the self-structuring fuzzy network calibration algorithm, the sample size used for network training affects the accuracy of the calibration network. Therefore, it is of great significance to improve the network calibration results by expanding the data sample size. Based on the Timoshenko beam theory, a non-linear relationship is shown between the measured strain and the node displacement. Therefore, the B-spline function is employed in this paper, in order to expand the sample size. It can realize the fitting of the relationship curve from a small amount of data, where the expansion of the sample capacity can be achieved by interpolation.

A B-spline curve of order p is defined as [25]

(12) $$C\left(u\right)=\overset{n}{\underset{i=0}{\sum }}N_{i,p}\left(u\right)P_{i},$$

where 0≤u≤1, $P_{i}$ is the control point, $N_{i,p}\left(u\right)$ is a B-spline basis function of order p, and the domain is a non-periodic node vector U. Its value is non-zero in the interval $\left[u_{i},u_{i+p+1}\right)$. The basic functions are calculated as

(13) $$N_{i,0}\left(u\right)=\{\begin{array}{c} 1\;\;\;\;\;\;\;\;\;\;u_{i}\leq u\leq u_{i+1},\\ 0\;\;\;\;\;\;\;\;\;\;\;\;\;\;\;\;\;otherwise, \end{array}$$

(14) $$N_{i,p}\left(u\right)=\frac{u-\;u_{i}}{u_{i+p}-\;u_{i}}N_{i,p-1}\left(u\right)+\frac{u_{i+p+1}-\;u}{u_{i+p+1}-\;u_{i+1}}N_{i+1,p-1}\left(u\right).$$

**Figure 2:**
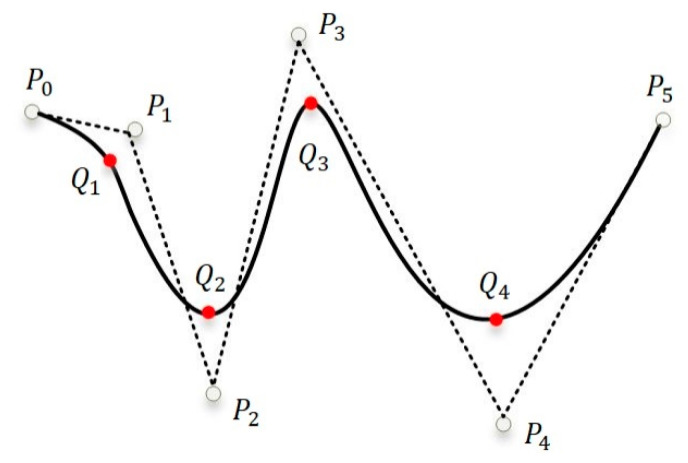
B-spline curve control polygon diagram.

A B-spline curve of order *p* is interpolated using a given set of points $\left\{Q_{k}\right\}\;\left(k=0,1,\cdots ,n\right)$. These points are the basic points of the curve, as shown in Figure 2.

After the first step of calibrating the reconstructed displacement, the data are composed of 6 measured strains and 8 kinematic variable error distribution values. Therefore, it can be regarded as a point in a 14-dimensional space $\left\{Q_{k}\right\}=\left\{{\left(\varepsilon_{1},\varepsilon_{2},\varepsilon_{3},\varepsilon_{4},\varepsilon_{5},\varepsilon_{6}\right)}_{k},{\left(\Delta u,\Delta v,\Delta w,\Delta \theta_{x},\Delta \theta_{y},\Delta \theta_{z},\Delta \theta_{oy},\Delta \theta_{oz}\right)}_{k}\right\}$ (k=0,1,⋯,n), where ε represents the measured strain. Each point $Q_{k}$ is assigned a parameter value ${\bar{u}}_{k}$, and the node vector $U=\left\{u_{0},u_{1},\cdots ,u_{m}\right\}$ is assigned a suitable value. Then, the following linear equations can be established:

(15) $$Q_{k}=C\left({\bar{u}}_{k}\right)=\overset{n}{\underset{i=0}{\sum }}N_{i,p}\left({\bar{u}}_{k}\right)P_{i}.$$

The parameter value is obtained with the method of uniform parameterization. Let ${\bar{u}}_{0}$ = 0 and ${\bar{u}}_{n}$ = 1. Then,

(16) $${\bar{u}}_{k}=\frac{k}{n}(k=1,2,\ldots n-1).$$

Then, we determine the node vector by taking the average method, and the intermediate node vector is obtained by Equation (17):

(17) $$u_{j+p}=\frac{1}{p}\overset{j+p-1}{\underset{i=j}{\sum }}{\bar{u}}_{i}\left(\;j=1,2,\cdots ,n-p\right).$$

Next, the control point $P_{i}$ can be obtained according to Formula (18), in order to fit the corresponding B-spline curve:

(18) $$\left[\begin{array}{c} P_{0}\\ P_{1}\\ \cdots \\ P_{7} \end{array}\right]={\left[\begin{array}{cc} \begin{array}{cc} N_{0,3}\left({\bar{u}}_{0}\right) & N_{1,3}\left({\bar{u}}_{0}\right)\\ N_{0,3}\left({\bar{u}}_{1}\right) & N_{1,3}\left({\bar{u}}_{1}\right) \end{array} & \begin{array}{cc} \cdots & N_{7,3}\left({\bar{u}}_{0}\right)\\ \cdots & N_{7,3}\left({\bar{u}}_{1}\right) \end{array}\\ \begin{array}{cc} \cdots & \cdots \\ N_{0,3}\left({\bar{u}}_{7}\right) & N_{1,3}\left({\bar{u}}_{7}\right) \end{array} & \begin{array}{cc} \cdots & \cdots \\ \cdots & N_{7,3}\left({\bar{u}}_{7}\right) \end{array} \end{array}\right]}^{-1}\left[\begin{array}{c} Q_{0}\\ Q_{1}\\ \cdots \\ Q_{7} \end{array}\right].$$

After obtaining the B-spline curve, let ${\bar{u}}_{k}$ take a value from 0 to 1 in a certain step and bring ${\bar{u}}_{k}$ into Equation (15), in order to obtain a large number of data points, $\left\{Q_{k}^{\prime }\right\}$, to achieve data expansion.

### 3.3. The Second Step of Calibration: Self-Structuring Fuzzy Network Calibration

**Figure 3:**
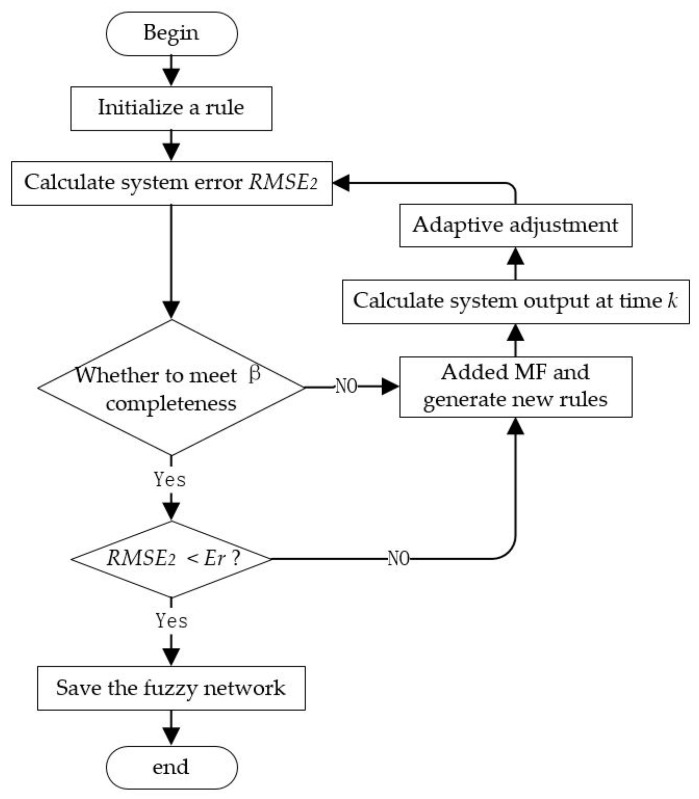
Flowchart of self-structuring fuzzy network algorithm.

In view of the reconstruction error caused by sensor installation and subsequent measurement, we use the SSFN method to calibrate the error in this paper. The membership function (MF) and rules of the SSFN can be increased and adjusted independently, in order to improve the fuzzy system structure in the self-structuring fuzzy network. The SSFN algorithm undergoes the following three phases: (1) Adding MF and generating rules; (2) Adaptive follow-up of fuzzy rules; and (3) Saving the fuzzy network [26]. The algorithm flowchart is shown in Figure 3.

#### 3.3.1. Add MF and Generation Rules

(1) Error criterion

$RMSE_{2}$ is used to describe the system error, which is given as follows:

(19) $$RMSE_{2}=\sqrt{\frac{\overset{N}{\underset{k=1}{\sum }}{\left(y\left(k\right)-y_{d}\left(k\right)\right)}^{2}}{N}},$$

where y(k) represents the output value of the SSFN and $y_{d}\left(k\right)$ represents the node displacement after the first calibration. $E_{r}$ represents the error threshold in the training stage. If $RMSE_{2}>E_{r}$, it means that the MF needs to be increased.

(2) Completeness criteria

For any input variable $x_{j}\left(k\right)$ in the interval, at least one MF can be activated. The maximum value of membership degree $\mu_{m}\left(x_{j}\left(k\right)\right)$ cannot be less than a pre-set value β. If $\mu_{m}\left(x_{j}\left(k\right)\right)<\beta$, the MF needs to be increased; otherwise, it is not increased.

#### 3.3.2. Self-Adaptation Rules

Based on the $RMSE_{2}$ in the SSFN, the consequent parameter of the rule is adjusted. At the current time *k*, the specific expression of adjusting the follower $\alpha_{j}\left(k\right)$ of the *j*th rule is as follows:

(20) $$\Delta \alpha_{j}\left(k\right)=\gamma \cdot \mu_{j}\left(k-1\right)\cdot \left(r\left(k-1\right)-y\left(k\right)\right),$$

where $\mu_{j}\left(k-1\right)$ represents the activation degree of the jth rule at time k−1, r(k−1) represents the estimated displacement input into the network at the last moment, and y(k) is the node displacement after the second calibration step of the output of the SSFN at the current moment. The value of *γ* is artificially adjusted to change the speed of the rule’s self-adaptation process.

#### 3.3.3. Save the Rules to Get the Fuzzy Network

After many iterations, the error of the SSFN system tends to converge. If $RMSE_{2}<E_{r}$, it means that the SSFN system is stable and the fuzzy rule base has been formed.

**Figure 4:**
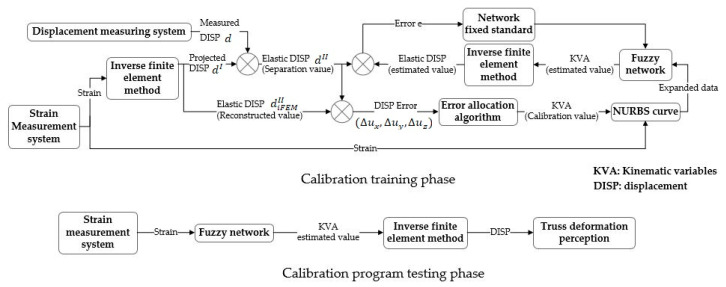
Block diagram of two-step calibration algorithm.

After expanding the strain and error distribution results, the extended samples are used to train the SSFN. The measured strain $\left(\varepsilon_{1},\varepsilon_{2},\varepsilon_{3},\varepsilon_{4},\varepsilon_{5},\varepsilon_{6}\right)$ is input into the SSFN, and the error distribution results $\left(\Delta u,\Delta v,\Delta w,\Delta \theta_{x},\Delta \theta_{y},\Delta \theta_{z},\Delta \theta_{oy},\Delta \theta_{oz}\right)$ are obtained. Then, the elastic displacement after calibration can be obtained. The error e can be calculated by the calibration displacement and the separated elastic displacement $d^{II}$. When e reaches a set standard, the existing SSFN is saved, completing the establishment of the fuzzy calibration network. The calibration algorithm block diagram is shown in Figure 4.

## 4. Experimental Examples

**Figure 5:**
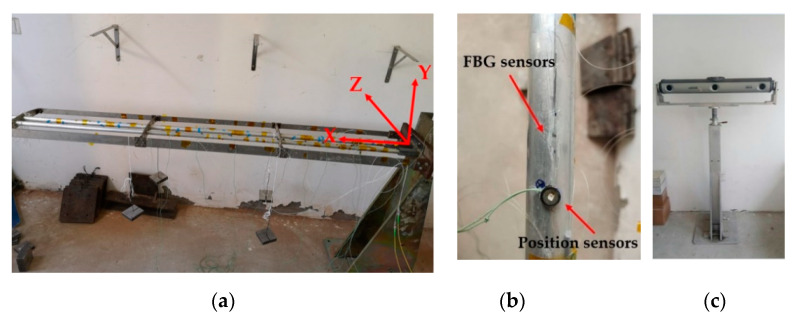
Experimental model of frame: (**a**) Frame model; (**b**) Displacement sensor; and (**c**) Displacement measuring instrument.

In this section, a three-element frame subject to static loading is used as an example, in order to demonstrate the efficiency and accuracy of the proposed method. The frame model is composed of two identical thin-walled beams and several thin-walled plates, while the model material is aluminum alloy. The length and thickness of each beam are 2 m and 1.5 mm, respectively, and the outer radius is 13 mm. The frame model can be divided into three parts, as shown in Figure 5a. The first element is near the fixed position, the second element is the middle part, and the third element is at the end. The length of each element is 660 mm.

In order to realize the deformation reconstruction of the frame, six fiber grating strain sensors are arranged on the surface of each element, in order to measure the surface strains [24]. The arrangement positions are shown in Table 1, where *x_k_* represents the relative position within the element and (*θ*,*β*) indicates that the sensor is placed at a circumferential angle. In the experiment, the strain data were obtained from a strain measurement system, which was composed of Fiber Bragg Grating (FBG) strain sensors (FBG; os1100, Micron Optics, Atlanta, GA, USA) and the FBG interrogator (Optical Sensing Instrument; Si 155, Micron Optics, Atlanta, GA, USA).

**Figure 6:**
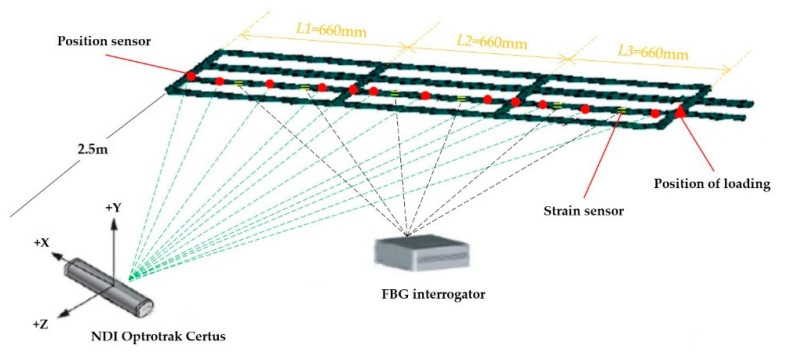
Experimental system.

Displacement measurements were performed at different locations along the beam with position sensors (see Figure 5b). The infrared light captured by the position sensor was detected by a three-dimensional dynamic displacement measurement instrument (NDI Optrotrak Certus, NDI, Canada; shown in Figure 5c), and the deformation of the frame was measured by the position sensor. The accuracy of NDI was 0.1 mm within its measurement range. The positions of the position sensors $\left(x_{m},y_{m},z_{m}\right)$ are shown in Table 2. The sensors numbered 5, 11, and 16 were used to measure the displacement of the end of each element, and the sensors numbered 3, 8, and 14 corresponded to the internal node positions of the three elements, respectively. When the strain measurement system collected strain data, the NDI collected displacement information at the same time. The entire experimental system is shown in Figure 6.

**Figure 7:**
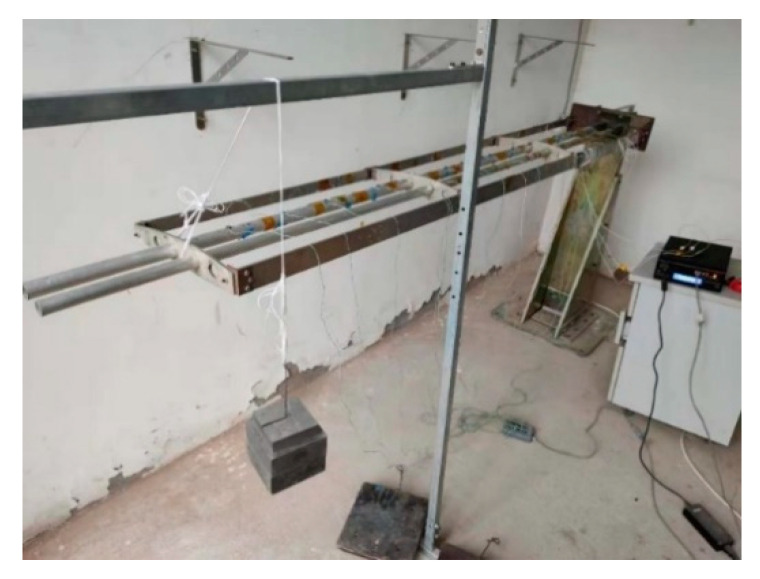
Model loading.

The error calibration adopted the principle of separately calibrating each element. A coordinate system was established, as shown in Figure 5a. At the end of the third element, static loading was performed diagonally upward (see Figure 6 and Figure 7). The loaded weights under different working conditions are shown in Table 3. The data from working conditions (1–8) were used to train the SSFN, and the data from working conditions (9–16) were used to verify the calibration accuracy of the SSFN. In working conditions (1–8), the $d^{I}$ and $d_{iFEM}^{II}$ of the node under each loading condition were obtained according to Equations (6) and (7). Combined with the displacement d measured by displacement measuring instrument, the elastic displacement $d^{II}$ was obtained using Equation (8). According to the Formula (9), the reconstruction error of elastic displacement $\left(\Delta u_{x},\Delta u_{y},\Delta u_{z}\right)$ was calculated using the $d^{II}$ and $d_{iFEM}^{II}$ values. Then, the errors were distributed to the six kinematic variables of the node, through the error distribution algorithm, in order to achieve the first step of calibration. The strain and error distribution data of each element were obtained under eight working conditions. The non-uniform rational B-spline interpolation algorithm was used to expand the 8 sets of data to 251 sets of data, providing a data set for SSFN training.

The working conditions (9–16) in Table 3 were used to verify the calibration accuracy of the trained SSFN. Inputting the measured strain into the trained SSFN, the kinematic variables after the second step calibration could be obtained. Then, the elastic displacements after two-step calibration were obtained by the inverse FEM and the projected displacement was added in order to obtain the final displacement calibration value of the node. The displacement at any point could be obtained through the node displacement and shape function (see Appendix A). In practical applications, the SSFN obtained by training can be used to perform calibration. This process can be performed 1000 times per second, from input strain to acquiring displacement after calibration.

In order to evaluate the calibration effect, the RMSE and relative root mean square error (RRMSE) were used as the measurement error indices. Their expressions are as follows:

(21) $$RMSE_{3}=\;\sqrt[{2}]{\frac{\overset{n}{\underset{i=1}{\sum }}{\left(disp^{NDI}\left(x_{i}\right)-disp^{modify}\left(x_{i}\right)\right)}^{2}}{n},}$$

(22) $$RRMSE=\frac{RMSE_{3}}{Max\left(disp^{NDI}\right)}\times 100\%,$$

where $disp\left(x_{i}\right)$ is the deformation displacement in one direction along the centroid axis. The superscript ‘NDI’ refers to the deformation values captured by the NDI; ‘modify’ refers to the displacement values after two-step calibration of the reconstruction deformation. RRMSE stands for relative root mean square error, which is the ratio of $RMSE_{3}$ to the maximum deformation value captured by NDI in the element. When the maximum loading (12 kg) was loaded, the deformation reconstruction values of the frame ends in the x, y, and z directions were 1.40 mm, 37.87 mm, and 19.81 mm, respectively. It can be seen that y and z were the main deformation directions. The calibration results of the first and second elements are shown in Table 4 and Table 5, respectively. In the table, RMSE is used as the error index in the x direction, while RRMSE is used as the error index in the y and z directions; the superscript ‘IFEM’ indicates the error between the inverse finite element method reconstruction displacement and the NDI capture displacement; while ‘modify’ indicates the error between the NDI capture displacement and the reconstruction displacement after the two-step calibration.

It can be seen from Table 4 that the maximum RMSE in the x direction was reduced from 0.35 to 0.18 mm after the first element calibration; the maximum RRMSE in the y direction was reduced from 12.62% to 5.57%; and the maximum RRMSE in the z direction was reduced from 13.77% to 7.68%. In the eight working conditions, the displacement errors in the x, y, and z directions of the first element after calibration were all reduced.

It can be discovered from Table 5 that after the second element was calibrated, the maximum RMSE in the x direction decreased from 0.59 to 0.25 mm, the maximum relative RMSE error in the y direction decreased from 5.92% to 2.07%, and the maximum relative RMSE error in the z direction decreased from 13.19% to 9.57%. Moreover, in all working conditions, the displacement errors in the x, y, and z directions of the second element after calibration were reduced.

**Figure 8:**
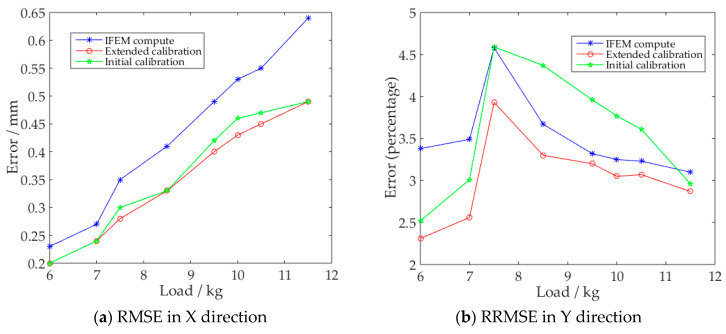

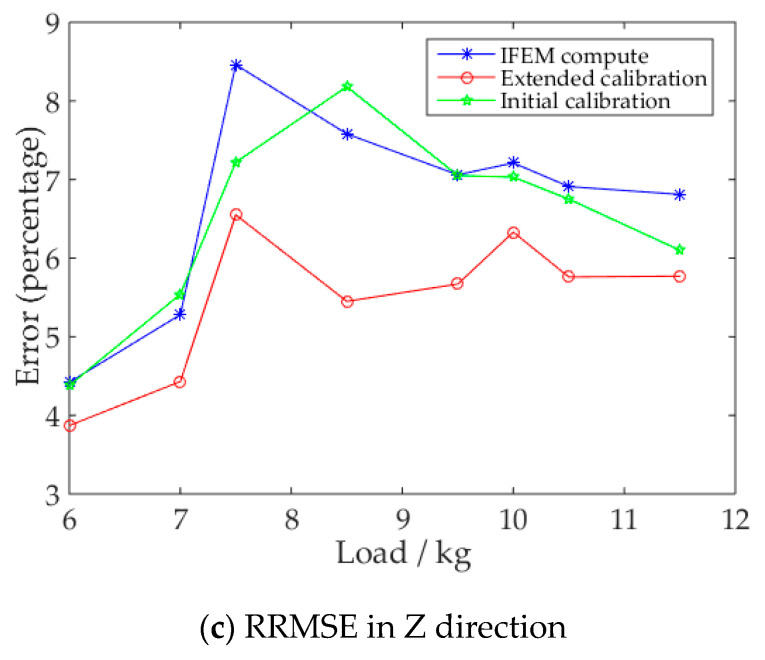
The overall x, y, and z direction errors of the three-element frame.

To explore the influence of the number of training samples on the calibration accuracy of the SSFN, the initial 8 sets of data and the expanded 251 sets of data were used to train the SSFN separately. The calibration results of the two networks are shown in Figure 8.

It can be seen from Figure 8b,c that for the reconstructed displacement errors in the y and z directions of the frame, the ‘initial calibration’ method achieved the effect of reducing errors in only a few working conditions; while according toFigure 8a–c, it can be seen that after using the ‘extended calibration’ method, all displacement errors were greatly reduced in the x, y, and z directions.

Based on the above calibration experiments on multi-element frame reconstruction displacements, the two-step calibration method proposed in this paper has good error calibration capabilities, with the sample expansion step making the calibration network more robust and accurate.

## 5. Conclusions

In actual engineering, sensor installation and strain measurement errors are inevitable, which affect the reconstruction accuracy of FEM. Therefore, in this paper, a two-step calibration method for FEM with small sample features was proposed in order to improve the reconstruction accuracy. Following experimental tests, the results showed that the reconstruction accuracy in the x, y, and z directions was significantly improved, regardless of whether the whole structure was calibrated or each element of a multi-element structure was individually calibrated. In particular, after calibrating the y-direction displacement of the first element, the maximum relative root mean square error was reduced from 12.62% to 5.57%. In addition, the experimental results showed that using the NURBS method to expand the sample data effectively improved the calibration effect of SSFN. Thus, this article provides an effective solution to the problem of small data samples.

## Figures and Tables

**Table 1:** Strain sensor locations.

$\mathrm{Axial}\;\mathrm{Position}\;x_{k}$	$0.3\;L_{1}$	$0.7\;L_{1}$	$0.3\;L_{2}$	$0.7\;L_{2}$	$0.3\;L_{3}$	$0.7\;L_{3}$
$\left(\theta_{1},\beta_{1}\right)$	(0,0)	(0,45)	(0,0)	(120,0)	(0,0)	(0,45)
$\left(\theta_{2},\beta_{2}\right)$	(120,0)	(−120,0)	(−120,0)	(−120,0)	(−120,0)	(−120,0)
$\left(\theta_{3},\beta_{3}\right)$	(−120,0)	(120,0)	(120,0)	(120,0)	(120,0)	(120,0)

**Table 2:** Position of position sensors.

Number	Position (mm)	Number	Position (mm)
1	(0,0,0)	9	(938.43,0,0)
2	(84.52,0,0)	10	(1017.57,0,0)
3	(179.42,0,0)	11	(1165.29,0,0)
4	(339.21,0,0)	12	(1232.87,0,0)
5	(525.25,0,0)	13	(1299.48,0,0)
6	(639.74,0,0)	14	(1474.19,0,0)
7	(780.89,0,0)	15	(1595.75,0,0)
8	(823.99,0,0)	16	(1725.31,0,0)

**Table 3:** Loading weight in different working conditions.

Number	Weight (kg)	Number	Weight (kg)
1	1.5	9	6
2	2.5	10	7
3	3.5	11	7.5
4	6.5	12	8.5
5	8	13	9.5
6	9	14	10
7	11	15	10.5
8	12	16	11.5

**Table 4:** Errors in the x, y, and z directions under different loads in the first element.

Load	6 kg	7 kg	7.5 kg	8.5 kg	9.5 kg	10 kg	10.5 kg	11.5 kg
${\mathrm{RMSE}}_{X}^{IFEM}$ (mm)	0.15	0.17	0.22	0.25	0.28	0.30	0.31	0.35
${\mathrm{RMSE}}_{X}^{modify}$(mm)	0.12	0.15	0.15	0.17	0.14	0.18	0.19	0.18
${\mathrm{RRMSE}}_{Y}^{IFEM}$	11.96%	12.62%	8.67%	9.78%	10.45%	10.37%	10.47%	10.74%
${\mathrm{RRMSE}}_{Y}^{modify}$	5.23%	5.57%	4.12%	2.20%	4.92%	1.78%	1.83%	2.43%
${\mathrm{RRMSE}}_{Z}^{IFEM}$	12.58%	12.74%	13.77%	13.22%	13.03%	12.88%	13.02%	12.79%
${\mathrm{RRMSE}}_{Z}^{modify}$	6.44%	7.86%	7.68%	9.01%	12.5%	8.32%	7.90%	11.4%

**Table 5:** Errors in the x, y, and z directions under different loads in the second element.

Load	6 kg	7 kg	7.5 kg	8.5 kg	9.5 kg	10 kg	10.5 kg	11.5 kg
${\mathrm{RMSE}}_{X}^{IFEM}$ (mm)	0.21	0.25	0.32	0.38	0.45	0.49	0.51	0.59
${\mathrm{RMSE}}_{X}^{modify}$(mm)	0.15	0.19	0.20	0.23	0.31	0.31	0.32	0.25
${\mathrm{RRMSE}}_{Y}^{IFEM}$	3.52%	2.99%	5.92%	4.21%	3.25%	3.11%	2.93%	2.56%
${\mathrm{RRMSE}}_{Y}^{modify}$	1.31%	1.18%	2.07%	0.87%	0.87%	0.95%	1.21%	1.49%
${\mathrm{RRMSE}}_{Z}^{IFEM}$	7.31%	6.30%	13.19%	11.59%	10.70%	10.67%	10.50%	9.97%
${\mathrm{RRMSE}}_{Z}^{modify}$	6.07%	3.69%	9.57%	7.41%	9.77%	10.16%	8.71%	8.85%

## Appendix A

According to the inverse finite element method proposed by Gherlone et al., the relationship expression of strain displacement can be obtained for any element of a structure:

(A1) KU=F,

where $K=\overset{6}{\underset{k=1}{\sum }}w_{k}L^{e}\left[B_{x}^{T}\left(x\right)B_{k}\left(x\right)\right]$, which is determined by the measurement position of the strain and is independent of the strain data; $F=\overset{6}{\underset{k=1}{\sum }}w_{k}L^{e}\left[B_{x}^{T}\left(x\right)e_{k}^{\varepsilon }\right]\;\left(k=1,\ldots ,6\right)$ is determined by the measured surface strain; U is the displacement of the first and last ends of the beam; $x\left(0\leq x\leq L^{e}\right)$ represents the coordinates of the cross-section where the strain is measured; $w_{k}\left(k=1,\ldots ,6\right)$ represents the gravity weighting coefficient; and $L^{e},\;B\left(x\right)$, and $e^{\varepsilon }$ represent the element length, deformation function, and actual measured section strain of the element, respectively. Therefore, for the unknown node deformation, it can be obtained for any cross-sectional strain; that is, $U=K^{-1}F$. For any certain unit, $K^{-1}$ remains unchanged and does not change with the change of working conditions.

According to the expression for F and Formula (A1), the relationship between the strain at any point in an element and the node displacement can be determined. For any point in the element, the displacement can be determined by the node displacement and the shape function:

(A2) $$u\left(\xi \right)=\underset{i=1,2}{\sum }L_{i}^{\left(1\right)}\left(\xi \right)u_{i},$$

(A3) $$v\left(\xi \right)=\underset{i=1,2}{\sum }L_{i}^{\left(1\right)}\left(\xi \right)v_{i}-\underset{i=1,r,2}{\sum }N_{j}^{\left(3\right)}\left(\xi \right)\theta_{zj},$$

(A4) $$w\left(\xi \right)=\underset{i=1,2}{\sum }L_{i}^{\left(1\right)}\left(\xi \right)w_{i}-\underset{i=1,r,2}{\sum }N_{j}^{\left(3\right)}\left(\xi \right)\theta_{yj},$$

(A5) $$\theta_{x}\left(\xi \right)=\underset{i=1,r,2}{\sum }L_{i}^{\left(1\right)}\left(\xi \right)\theta_{xi},$$

(A6) $$\theta_{y}\left(\xi \right)=\underset{j=1,r,2}{\sum }L_{j}^{\left(2\right)}\left(\xi \right)\theta_{yj},$$

(A7) $$\theta_{y}\left(\xi \right)=\underset{j=1,r,2}{\sum }L_{i}^{\left(2\right)}\left(\xi \right)\theta_{zj},$$

where u(ξ), v(ξ), w(ξ), $\theta_{x}\left(\xi \right)$, $\theta_{y}\left(\xi \right)$, and $\theta_{z}\left(\xi \right)$ represent the displacement components at any point in the element; $\xi =\left(\frac{2x}{L^{e}}-1\right)\in \left[-1,1\right]$ is a dimensionless coordinate indicating the position of the displacement point within the element; $\underset{i=1,2}{\sum }L_{i}^{\left(1\right)}\left(\xi \right)$ and $\underset{i=1,r,2}{\sum }L_{i}^{\left(2\right)}\left(\xi \right)$ represent linear and quadratic Lagrange polynomial shape functions, respectively; $\underset{i=1,r,2}{\sum }N_{i}^{\left(3\right)}\left(\xi \right)$ represents the cubic Lagrange polynomial shape function; $\underset{i=1,2}{\sum }u_{i}$, $\underset{i=1,2}{\sum }v_{i}$, $\underset{i=1,2}{\sum }w_{i}$, $\underset{i=1,2}{\sum }\theta_{xi}$, $\underset{i=1,2}{\sum }\theta_{yi}$, and $\underset{i=1,2}{\sum }\theta_{zi}$ represent the displacement components of the nodes at both ends in the element; and $\underset{i=1,r,2}{\sum }\theta_{yi}$ and $\underset{i=1,r,2}{\sum }\theta_{zi}$ represent the displacement components of the two nodes and the intermediate points in the element.
